# Impact of a Blend of Microencapsulated Organic Acids and Botanicals on the Microbiome of Commercial Broiler Breeders under Clinical Necrotic Enteritis

**DOI:** 10.3390/ani13101627

**Published:** 2023-05-12

**Authors:** Dana K. Dittoe, Casey N. Johnson, James A. Byrd, Steven C. Ricke, Andrea Piva, Ester Grilli, Christina L. Swaggerty

**Affiliations:** 1 Animal Science Department, University of Wyoming, 1000 E University Ave., Laramie, WY 82071, USA; ddittoe@uwyo.edu; 2 U.S. Department of Agriculture, Agricultural Research Service, Southern Plains Agricultural Research Center, College Station, TX 77845, USA; casey.n.johnson@usda.gov (C.N.J.); allen.byrd2@usda.gov (J.A.B.II); 3 College of Agricultural and Life Sciences, University of Wisconsin, 1933 Observatory Dr., Madison, WI 53706, USA; sricke@wisc.edu; 4 DIMEVET, Department of Veterinary Medical Sciences, University of Bologna, Via Tolara di Sopra 50, Ozzano Emilia, 40064 Bologna, Italy; andrea.piva@unibo.it (A.P.); ester.grilli@unibo.it (E.G.); 5 Vetagro S.p.A., Via Porro 2, 42124 Reggio Emilia, Italy; 6 Vetagro Inc., 17 East Monroe St. Suite 179, Chicago, IL 60603, USA

**Keywords:** *Clostridium perfringens*, feed additive, microbiome, microencapsulated organic acids and botanicals, necrotic enteritis

## Abstract

**Simple Summary:**

Necrotic enteritis, a secondary gastrointestinal (GIT) infection in poultry, is a costly burden to the commercialized poultry industry, impacting the welfare of broiler chickens as well as production gains. Many feed supplements have been proposed to alleviate the burden of necrotic enteritis in poultry with varied success. A promising supplement, a microencapsulated blend of organic acids and botanicals, has been demonstrated to modulate the immune system and the gastrointestinal microbial ecology in healthy birds. Therefore, we aimed to test this supplement in the feed of broiler breeder chickens infected with necrotic enteritis. To determine if the supplement improved the microbial ecology of the GIT, where necrotic enteritis resides, the GIT microbiota were elucidated using microbiome sequencing. There were minimal differences in the microbiota diversity; however, the core microorganisms of birds fed the supplement consisted of *Lactobacillus* and *Clostridiaceae*. The supplemented-fed birds also had a higher abundance of normal microbiome inhabitants, namely, *Actinobacteriota*, *Bacteroidota*, and *Verrucomicrobiota*, than those not fed the supplement. Therefore, the supplementation of a microencapsulated blend supported beneficial and core microorganisms, potentially improving the bird’s response to NE.

**Abstract:**

Previously, the supplementation of a microencapsulated blend of organic acids and botanicals improved the health and performance of broiler breeders under non-challenged conditions. This study aimed to determine if the microencapsulated blend impacted dysbiosis and necrotic enteritis (NE) in broiler breeders. Day-of-hatch chicks were assigned to non-challenge and challenge groups, provided a basal diet supplemented with 0 or 500 g/MT of the blend, and subjected to a laboratory model for NE. On d 20–21, jejunum/ileum content were collected for microbiome sequencing (n = 10; V4 region of 16S rRNA gene). The experiment was repeated (n = 3), and data were analyzed in QIIME2 and R. Alpha and beta diversity, core microbiome, and compositional differences were determined (significance at *p* ≤ 0.05; Q ≤ 0.05). There was no difference between richness and evenness of those fed diets containing 0 and 500 g/MT microencapsulated blend, but differences were seen between the non-challenged and challenged groups. Beta diversity of the 0 and 500 g/MT non-challenged groups differed, but no differences existed between the NE-challenged groups. The core microbiome of those fed 500 g/MT similarly consisted of *Lactobacillus* and *Clostridiaceae.* Furthermore, challenged birds fed diets containing 500 g/MT had a higher abundance of significantly different phyla, namely, *Actinobacteriota*, *Bacteroidota*, and *Verrucomicrobiota*, than the 0 g/MT challenged group. Dietary supplementation of a microencapsulated blend shifted the microbiome by supporting beneficial and core taxa.

## 1. Introduction

Necrotic enteritis (NE), often a secondary infection to coccidiosis (*Eimeria* spp.) caused by the opportunistic pathogen *Clostridium perfringens* [1], was estimated to globally cost the poultry industry USD 2 billion annually [2]. However, with the removal of antibiotic growth promoters (AGPs) from commercial poultry diets [3], there has been an associated increase in the incidence of NE in broiler flocks [4,5]. As such, more recent estimations (2015) of the economic burden of NE are closer to USD 6 billion annually across the globe [6].

The use of AGPs effectively reduced the incidence of NE by limiting the prevalence of coccidiosis and manipulating the intestinal microbiome [7,8]. Effective AGP alternatives would need to elicit similar responses. Alternative feed additives such as probiotics, phytogenics (essential oils, plant-derived compounds), and organic acids are promising candidates [1]. However, these alternatives’ efficacy in mitigating NE is inconsistent and merits further investigation [9]. These inconsistencies could result from numerous factors such as inclusion level, mode of action, or the location where the alternative is available to the host [1].

Microencapsulation using a lipid matrix is a promising technology that could allow for the improved efficacy of feed supplements. In recent years, with the advancement in technology, the microencapsulation of feed additives has allowed additives to bypass the crop and gizzard, reaching the lower small intestines [10], where nutrient digestion occurs [11,12]. As such, recent research investigating the efficacy of AviPlus^®^ P (Vetagro S.p.A., Reggio Emilia, Italy), a microencapsulated blend of organic acids (25% citric and 16.7% sorbic) and botanicals (1.7% thymol and 1% vanillin), has demonstrated promising results on modulating the gastrointestinal tract (GIT) of broilers [13] and improving intestinal integrity and the inflammation of swine [14]. In addition, during a clinical infection model of NE, supplementation with the microencapsulated blend of organic acids and botanicals resulted in a decrease in mortality (*p* = 0.004), lower lesion scores (*p* = 0.006), and tended to improve body weight at d 20–21 (*p* = 0.19) compared to the control-fed birds by modulating the T cell receptor, TNF, and NF-kB signaling pathways and subsequent cytokine responses [15].

In a continuation with the efforts to determine the efficacy of a microencapsulated blend of organic acids and botanicals (AviPlus^®^ P) as an alternative feed amendment [13,14,15,16,17], the objective of the current study was to: (1) elucidate the microbiome response to a microencapsulated blend of organic acids and botanicals in the lower small intestines during a clinical manifestation of NE; (2) describe the relationship of NE severity with the microbiome diversity and richness in the lower GIT, with and without the supplementation of a microencapsulated blend of organic acids and botanicals in the diet; and (3) determine the core microbiome of the lower GIT in response to the supplementation of a microencapsulated blend of organic acids and botanicals. Therefore, broiler breeders were infected with *Eimeria* spp. (d 14) and, subsequently, with *Clostridium perfringens* (d 17–19) to induce NE. On d 20 to 21, the digesta from the lower small intestines was collected for microbiome analyses via sequencing the V4 region of the 16S rRNA gene.

## 2. Materials and Methods

### 2.1. Husbandry

The current experiment was repeated three independent times (trials = 3), with chicks being sourced from different flocks for each trial to ensure the reproducibility of results. At the onset of each trial, on day-of-hatch, by-product male broiler breeder chicks (*Gallus gallus*) were sourced from a commercial hatchery, placed in floor pens (3 × 3 m) with fresh pine shavings, and provided feed, water, and supplemental heat ad libitum. Male chicks were randomly assigned to pens (n = 23–26; one pen/treatment/experimental replicate) and provided a control-basal diet or a supplemented basal diet. The basal diet, an unmedicated corn and soybean meal starter diet, was formulated according to industry standards to meet or exceed the established nutrient requirements [18]. The supplemented diet contained 500 g/MT of a microencapsulated blend of citric (25%) and sorbic (16.7%) acids, thymol (1.7%), and vanillin (1.0%) (AviPlus^®^ P, Vetagro S.p.A., Reggio Emilia, Italy). From the onset of the experiments, no medication or other therapeutic interventions were administered. In accordance with the Guide for the Care and Use of Agricultural Animals in Research and Training [19], the care and handling of poultry was ethically conducted in the presence of an on-site veterinarian. All bird studies were under the approved experimental procedures outlined in protocol number 2019-002 and were approved by the USDA/ARS Institutional Animal Care and Use Committee operating under the Animal and Plant Health Inspection Service establishment number 334299.

### 2.2. Clostridium Perfringens Preparation

In all independent trials, a total of four field isolates of wild-type *C. perfringens* (type A) were obtained from active and confirmed outbreaks of NE in Georgia (two isolates), Texas (one isolate), and Virginia (one isolate). Prior to the onset of the experiment, frozen-stock isolates were cultured separately in thioglycollate broth (Becton Dickinson Co., Sparks, MD, USA) for 12 h anaerobically. Fresh cultures were then combined to yield the challenge stock, as previously described [20].

### 2.3. Necrotic Enteritis Model

On d 14, chicks were orally administered a 2× dose of a commercially available coccidiosis vaccine (1 mL, Coccivac^®^-B52; Merck Animal Health, Kenilworth, NJ, USA). On d 17–19, birds were additionally challenged orally (3 mL) with a stock culture containing the cocktail of the four *C. perfringens* strains (10^7^ colony forming units (CFU)/mL) to induce NE. For all three experimental replicates, on d 20 or 21, chickens were euthanized by cervical dislocation and necropsied, where birds were scored for intestinal lesions, and the digesta of the jejunum/ileum was collected for microbiome sequencing (see next section for additional details). The aseptically collected digesta samples (6–10/group) were then shipped on dry ice to the Center for Food Safety at the University of Arkansas (Fayetteville, AR, USA) to prepare a DNA library for microbiome sequencing. Upon arrival at the Center for Food Safety, samples were stored at −20 °C until DNA extraction could be conducted.

### 2.4. Scoring Necrotic Enteritis Lesions

As described by Prescott, NE severity was evaluated by scoring gross lesions of the jejunum and ileum of the small intestines [21]. Regardless of the trial (n = 3), one person blindly scored the intestines to reduce bias across the experiment, and these data are published [15], but, for clarity, the scale is also provided herein. Lesions were scored on a scale ranging from 0 to 4: (0) normal healthy tissue with no gross lesions; (1) thin-walled or fragile tissue with ashen appearance; (2) thin-walled, focal necrosis, grey in appearance with small gas production; (3) thin-walled, considerable patches of necrosis, prolific gas production within intestines, small areas of blood; (4) acute widespread necrosis, pronounced hemorrhages and vast gas production.

### 2.5. DNA Extraction

The digesta samples were allowed to thaw on wet ice, and 200 mg of the digesta was aliquoted to a sterile microcentrifuge tube. The DNA of the 200 mg was then extracted according to the standard protocol of the QIAamp Fast DNA Stool Mini Kit and eluted in 100 μL of AE Buffer (Qiagen, Hilden, Germany). The DNA concentration was determined using a Nanodrop™ 1000 spectrophotometer (Thermo Fisher Scientific, Waltham, MA, USA). Samples with DNA concentrations under 15 ng/μL were not diluted; however, those exceeding 15 ng/μL were diluted to 10 ng/μL in Buffer AE (Qiagen, Hilden, Germany).

### 2.6. Library Preparation

Using dual-indexed primers, including unique eight nucleotide barcode sequences, developed by Kozich et al. [22], the V4 region of the 16S region was amplified using a high-fidelity polymerase (Accuprime Pfx DNA polymerase, Thermo Fisher Scientific, Waltham, MA, USA). Amplified products were verified using gel electrophoresis. Verified amplified products were normalized to equimolar and equal volumes (20 μL) using a SequalPrep™ Normalization kit (Life Technologies, Carlsbad, CA, USA). Following this, 5 μL of the normalized samples was aliquoted to one microcentrifuge tube (pool) to create the final library. Library concentrations were determined using both a KAPA library quantification kit for Illumina platforms (Kapa Biosystems, Inc., Wilmington, MA, USA) and with a Qubit 4 fluorometer using a 1× High Specificity Assay Kit (Invitrogen, Carlsbad, CA, USA). Amplicon product size was verified and assessed using an Agilent 2100 Bioanalyzer (Agilent, Santa Clara, CA, USA). The library and PhiX Control v3 (Illumina, San Diego, CA, USA) were diluted to 20 nM in HyB buffer and denatured in 0.2 N of fresh NaOH for a final concentration of 6 pM. The diluted library was then combined with PhiX for a final PhiX concentration of 20% (wt/vol), loaded into a MiSeq V2 500 cycle sequencing cartridge, and sequenced on an Illumina MiSeq (Illumina, San Diego, CA, USA). Resulting sequences (fastq files) were downloaded from Illumina BaseSpace and uploaded to the NCBI Sequence Read Archive (PRJNA899529) and GitHub https://github.com/RickeLab-UW/Microencapsulated-Blend-on-Broiler-Intestinal-Microbiota (accessed on 3 May 2023).

### 2.7. Bioinformatic Analyses

Raw sequencing data were demultiplexed and downloaded from Illumina BaseSpace (Illumina, San Diego, CA, USA). Demultiplexed sequences were uploaded into QIIME2 (quantitative insights into microbial ecology; 2021.11) using the paired-end demultiplexed version of Casava 1.8 (via QIIME 2 tools import) [23]. Imported demultiplexed sequences were then filtered and denoised using DADA2 (divisive amplicon denoising algorithm) (via q2-dada2) [24]. The ASVs were assigned using mafft with a rooted phylogenetic tree being produced using fasttee2 (via q2-phylogeny) [25]. Prior to taxonomic identification, the ASVs identified as mitochondria and chloroplast were filtered from the tables and sequences using taxonomy-based filtering via q2-taxa. They were, subsequently, excluded from all statistical analyses. Due to the evolution of mitochondria and chloroplast from bacterial origins, these eukaryotes contained 16S rRNA, and the subsequent sequences must have been removed prior to the interpretation of bacterial populations.

The treatments (NE infection and dietary supplementation) were applied as a 2 × 2 factorial designs; however, due to the limitations of advanced statistical models and post hoc analyses, the main effect of overall treatment (0 g/MT CON, 500 g/MT CON, 0 g/MT NE, 500 g/MT NE) was evaluated. As well as the interaction of treatment and trial (block effect) using ANOVA and ADONIS for alpha and beta diversity metrics via q2-longitudinal were determined [26,27]. The interaction of treatment and trial was performed to determine if the random variable, namely, trial (block effect), had a significant effect on the diversity results. Pairwise comparisons between treatment groups for alpha diversity metrics, namely, Shannon’s Entropy and Pielou’s Evenness, were completed using Kruskal–Wallis [28,29]. Pairwise comparisons between the beta diversity metrics, namely, Jaccard Distance, and Weighted Unifrac, of treatment groups were performed using ANOSIM (analysis of similarities) [30,31,32]. Using ANCOM, the differentially abundant taxa were determined between treatment groups (via q2-composition) [33].

Additionally, the lesion score data were incorporated into the metadata file. A linear mixed effect model was utilized to determine if a relationship existed between the richness and evenness of the microbiome and the severity of NE as determined by lesion scores (via q2-longitudinal). In the linear mixed model, trial was treated as the random effect. The main effects were considered significant at *p* ≤ 0.05 and pairwise differences at Q ≤ 0.05.

Taxonomic identification of the ASVs was performed using the sk-learn Bayesian algorithm (95% confidence interval) against SILVA full-length sequences (MD5: b8609f23e9b17bd4a1321a8971303310) [34,35], via q2-feature-classifier [36]. The feature table, taxonomy, and rooted-tree phylogenetic were imported into R Studio (R Studio 1.4.1103; R 4.2.1), where a heatmap (ggplot2) at the genus level was generated, and the core microbiome was determined (phyloseq; microbiome utilities) [37,38]. Core microbiome members were determined with a detection setting of 0.001 and a prevalence of more than 50%. All figures, with the exception of PCOA plots (QIIME2) and heatmaps (R Studio), were generated in Microsoft Excel (Microsoft, Redmond, WA, USA).

## 3. Results

### 3.1. Alpha Diversity and Its Relation to Lesion Scores

**Figure 1:**
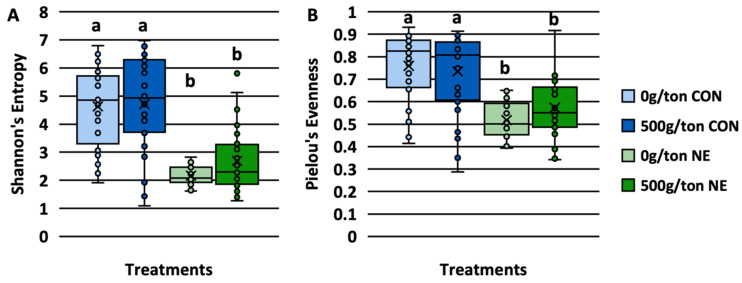
Shannon’s Entropy (**A**) and Pielou’s Evenness (**B**) of the microbiome of commercial broiler breeders fed diets containing a blend of microencapsulated organic acids and botanicals. Significant different means (×) are denoted with different connecting letters (a, b).

There was no interaction between treatment and trial on the richness of the microbiome, but there was a main effect of treatment on the richness (*p* < 0.05; Supplemental Table S1). Within the main effect of treatment, the richness of the digesta from birds infected with NE was significantly lower than those not infected (Q < 0.05; Figure 1A; Supplemental Table S2). However, there was no difference in richness between the supplemented diets within non-infected and infected birds (Q > 0.05). Although not statistically different, the mean richness was higher among the infected birds supplemented with 500 g/MT of a microencapsulated blend of organic acids and botanicals (Q > 0.05).

There was an interaction between treatment and trial (*p* = 0.045) but not a main effect of trial (*p* = 0.594) on the evenness of the community (Supplemental Table S1). Therefore, there was some interaction between treatment and trial but most likely due to the strong main effect of treatment (*p* < 0.001). The microbiome of the birds not infected with NE (CON) had a greater evenness than those infected (NE) (Q < 0.05; Figure 1B; Supplemental Table S2). As with richness, there was no difference in the addition of a microencapsulated blend of organic acids and botanicals on the evenness with either the non-infected or infected groups (Q > 0.05). However, those fed diets supplemented with a microencapsulated blend of organic acids and botanicals tended to have a greater evenness than those not supplemented when NE was induced (Q = 0.079).

**Figure 2:**
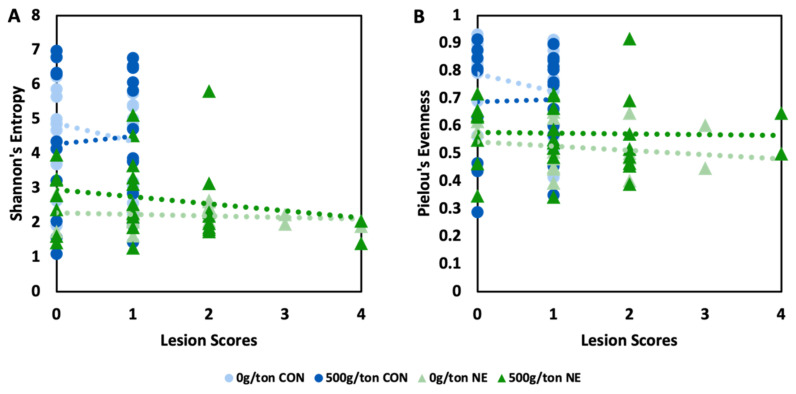
Relationship of lesions scores to the richness (**A**) and evenness (**B**) of the microbiome of commercial broiler breeders supplied supplemented diets containing a blend of microencapsulated organic acids and botanicals.

When investigating the relationship between the severity of NE as determined by the lesion scores, a linear mixed model was explored. The model produced a significant effect of treatment on both the richness and the evenness of the microbiome (Supplemental Table S3). However, there was no interaction between the treatments and lesion severity or a main effect of lesions on the richness and evenness of the microbiome composition (*p* > 0.05). Although there was no significant relationship, there were parallel relationships between richness and evenness and lesion scores of those with induced NE (Figure 2). The relationship of those with induced NE was almost parallel, with no change in richness and evenness as lesion scores increased. However, those without NE appeared to share a relationship (*p* > 0.05) with those fed diets containing 0 g/MT of a microencapsulated blend of organic acids and botanicals having a negative relationship and those fed diets containing 500 g/MT of a microencapsulated blend of organic acids and botanicals having a positive relationship between lesion scores and alpha diversity (Figure 2).

### 3.2. Beta Diversity Strongly Related to Supplement and Necrotic Enteritis Infection

**Figure 3:**
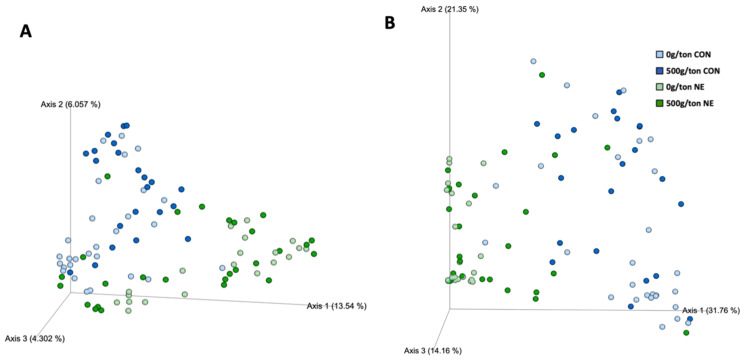
Abundance (**A**) and phylogenetic diversity (**B**) of the microbiome of commercial broiler breeders fed diets containing a blend of microencapsulated organic acids and botanicals.

There was a main effect of treatment and trial as well as their interaction on the abundance, and phylogenetic diversity of the microbiome of broiler breeders fed diets containing a microencapsulated blend of organic acids and botanicals (*p* <0.05; Supplemental Table S3). Within the treatment effect, there was a difference in both abundances and weighted phylogenetic distances between all treatments except those within the NE-induced group (0 g/MT and 500 g/MT NE; Q > 0.05; Figure 3; Supplemental Table S4). There was also a clear separation in abundances and weighted phylogenetic distances between those not infected (CON) and those infected (NE; Figure 3B; Supplemental Table S5).

### 3.3. Core Communities Shifted by Feed Additive and Infection

**Figure 4:**
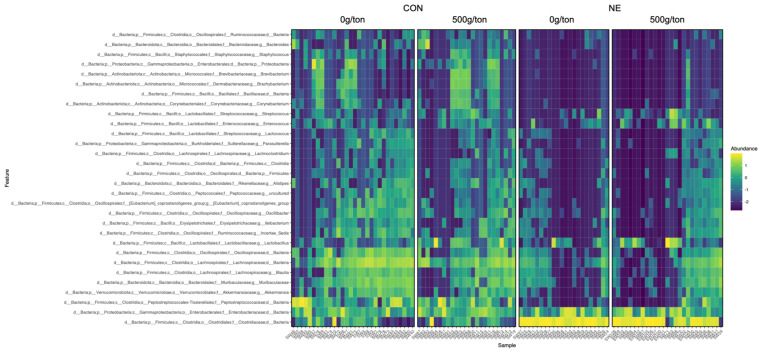
Distribution of the microbiome within the four treatment groups, control and NE enteritis induced birds fed diets supplemented with or without 500 g/MT of a blend of microencapsulated organic acids and botanicals.

To demonstrate the impact clinical NE-induced infection and supplementation of diets with 500 g/MT of a blend of microencapsulated organic acids and botanicals had on the prevalence of the microbiome members, a heatmap was produced (Figure 4). To note, there was a higher prevalence of genera belonging to the phyla Firmicutes, Actinobacteriota, and Bacteroidota among the digesta of the control birds compared to the NE induced populations. Regardless of infection, *Enterobacteriaceae* was highly prevalent in both populations. In the NE-induced population, the family of *Clostridiaceae* was highly prevalent. Looking at prevalence within the NE-induced chicks fed diets supplemented with 500 g/MT of a blend of microencapsulated organic acids and botanicals, the prevalence of genera within the phyla Firmicutes, Actinobacteriota, and Bacteroidota does appear to be restored compared to the non-supplemented NE group.

Although the heatmap allows the visualization of the impact of treatment on the members of the microbiome, it does not definitively determine the core members of the treatment groups. To determine the core members of the microbiome, core microbiome analyses were utilized, with core members being defined as being prevalent in 50% of samples within respective treatments. Of the non-supplemented control birds, the core microbiome consisted of 10 different amplicon sequence variants (ASV) belonging to the families: *Lachnospiraceae*, *Erysipelotrichaceae*, *Enterobacteriaceae*, *Sutterellaceae*, *Akkermansiaceae*, and *Peptostreptococcaceae* (Table 1). Six unique ASVs belonging to the families of *Lachnospiraceae*, *Clostridiaceae*, *Lactobacillaceae*, *Enterobacteriaceae*, and *Peptostreptococcaceae* were present in more than 50% of the control birds fed diets supplemented with 500 g/MT of microencapsulated organic acids and botanicals. In the NE-induced birds fed the control diets, only five unique ASVs were present in more than 50% of the birds. The core microbiome of the control-fed NE-induced birds consisted of members of the *Clostridiaceae* (4) and *Enterobacteriaceae* (1) families. Those fed diets containing the blend of microencapsulated organic acids and botanicals had seven unique ASVs identified as the core microbiome. Of those core members, six unique ASVs belonged to the family *Clostridiaceae* and one to *Enterobacteriaceae*.

Not many unique ASVs were considered core members across all four treatment groups. All groups shared one unique ASV belonging to the family of *Enterobacteriaceae*. Of the control, non-NE-induced birds, four unique ASVs were shared, belonging to the families *Peptostreptococcaceae*, *Lachnospiraceae*, and *Enterobacteriaceae*. In the NE-induced birds, the three ASVs belonged to the family *Clostridiaceae,* which were considered core members.

### 3.4. Relative Abundance of Taxa Impacted by Diet Supplementation and NE Infection

**Figure 5:**
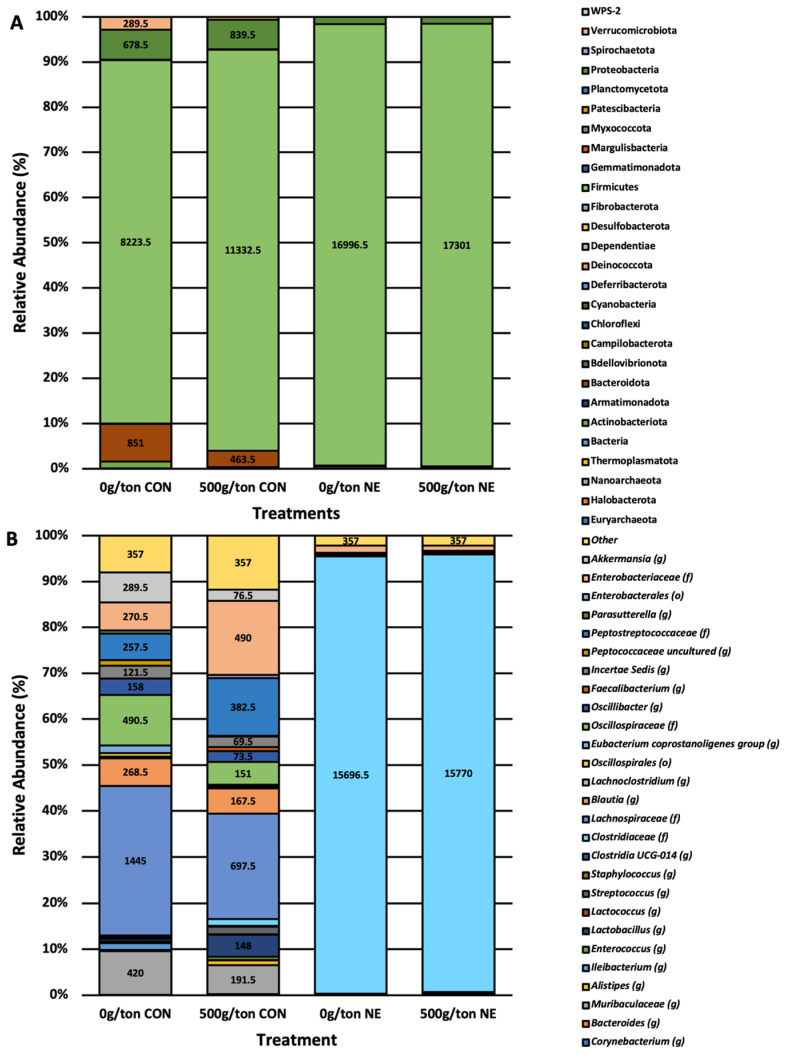
Taxonomic composition at the phylum (**A**) and genus (**B**) level of broiler breeders fed diets containing a blend of microencapsulated organic acids and botanicals. Significant different phyla, Actinobacteriota, Bacteroidota, Firmicutes, and Verrucomicrobiota, impacted by the supplementation of a blend of microencapsulated organic acids and botanicals (W = 24, 24, 26, 22; *p* < 0.05; Supplemental Figure S1). Significant different taxa at the genus level, *Clostridiaceae* and *Peptostreptococcaceae*, were impacted by the supplementation of a blend of microencapsulated organic acids and botanicals (W = 385, 369; *p* < 0.05; Supplemental Figure S2).

At the phyla level, the taxa most abundant were Firmicutes, Proteobacteria, and Bacteroidota, which comprised over 90% of the relative abundance of the microbiome (Figure 5A). Using analysis of communities of the microbiome (ANCOM), it was determined that Actinobacteriota, Bacteroidota, Firmicutes, and Verrucomicrobiota were impacted by the supplementation of a blend of microencapsulated organic acids and botanicals (W = 24, 24, 26, 22; *p* < 0.05; Supplemental Figure S1). The abundance of Actinobacteriota, Bacteroidota, and Verrucomicrobiota was decreased when NE was induced; however, the supplementation of 500 g/MT of a microencapsulated blend of organic acids and botanicals did appear to numerically increase these phyla compared to the non-supplemented groups. Likewise, those infected with *Eimeria spp.* and *C. perfringens* had higher levels of Firmicutes (Supplemental Figure S1).

At the genus level, there was a clear separation between the groups not infected (CON) and those with induced NE (NE; Figure 5B). Those not infected had a diverse taxonomic profile primarily comprised *Enterobacteriaceae*, *Peptostrptococcaceae*, *Incertia sedis*, *Oscillospiraceae*, *Blautia*, *Lachnospiraceae*, *Lactobacillus*, and *Muribaculaceae* when examined at the genus level. However, within those infected and NE was induced (NE), the microbiome composition was predominantly composed of *Clostridiaceae* and *Enterobacteriaceae*. The significantly different taxa at the genus level, as determined by ANCOM, were *Clostridiaceae* and *Peptostreptococcaceae* (W = 385, 369; *p* < 0.05; Supplemental Figure S2). The levels of *Clostridiaceae* were elevated in those with clinical NE-induced, while the levels of *Peptostreptococcaceae* were decreased in the same group. However, those supplemented with 500 g/MT of a microencapsulated blend of organic acids and botanicals (median of 509 features) had a higher level of *Peptostreptococcaceae* compared to those not supplemented with a microencapsulated blend of organic acids and botanicals (median of 1 feature).

## 4. Discussion

Understanding the impact of feed supplements on the microbiome of poultry is an important component in delineating the benefits of these additives, as the microbiome of the poultry GIT is directly impacted by feed supplementation and, subsequently, improves performance and disease resistance [39]. Therefore, many of the feed supplements utilized in the poultry industry that serve as antibiotic alternatives do so by directly impacting the microbiome of poultry. Recently, we have investigated the effect of a microencapsulated blend of organic acids and botanicals on broiler chickens on the performance, health, and microbiome under non-challenged conditions [13,16,17]. However, to determine the efficacy of the supplement in industry settings, we induced clinical NE in broiler breeder chickens fed diets supplemented with a microencapsulated blend of organic acids and botanicals. Supplementing the microencapsulated blend of organic acids and botanicals at 500 g/MT could reduce clinical signs of NE by modulating key specific immune-related pathways [15]. In continuation with these efforts, the current study aimed to determine if clinical signs of NE were improved by manipulating the microbiome of the lower small intestines (jejunum/ileum).

### 4.1. Lesion Scores Impact on Richness and Evenness

Previous research investigating the effect of supplementing broiler diets with a microencapsulated blend of organic acids and botanicals [13] showed no effect of diets on the alpha diversity of the jejunum and ileum (Shannon’s Diversity and Pielou’s Evenness). Instead, differences were driven by compartmental differences in the small intestines (jejunum and ileum). Pham et al. [40] also reported the lack of differences in the richness and evenness of the cecal microbiome when NE-challenged (sub-clinical infection) broiler chickens were fed diets supplemented with a microencapsulated blend of 4% thyme, 4% carvacrol, 0.5% hexanoic acid, 3.5% benzoic acid, and 0.5% butyric acid. The current study induced NE among broiler breeders fed 0 and 500 g/MT of the microencapsulated blend of organic acids and botanicals and demonstrated differences between treatment groups. However, as seen in the previous study, richness and evenness were less driven by the dietary treatments. The induction of clinical NE highly drove the differences. Therefore, as seen previously by Feye et al. [13], the dietary inclusion of a microencapsulated blend of organic acids and botanicals supplement did not appear to alter the richness and evenness of the microbiome of the small intestines.

In addition, the current study demonstrated the relationship of NE severity to the richness and evenness of the microbiome. Although there was no interaction between treatment and lesion score, the treatment had a significant effect. There was a parallel negative relationship between lesion score severity and richness and evenness of those induced with clinical NE. Those treated as controls (non-infected) did demonstrate relationships with the lesion scores (0 to 1). In contrast, those fed 0 g/MT had a negative association with lesion scores as the richness and evenness narrowed as the lesion scores reached 1. In comparison, those fed 500 g/MT and uninfected exhibited a positive relationship where the richness and evenness increased or stabilized as the lesion score increased. The current study presented evidence that the microbiome was correlated to the severity of lesion scores and that the supplementation of a microencapsulated blend of organic acids and botanicals had the potential to maintain or stabilize the richness and evenness of the community. However, it is important to note that the dietary treatments 0 and 500 g/MT microencapsulated blend of organic acids and botanicals behaved similarly when under clinical NE conditions.

### 4.2. Treatment and Infection on Abundance and Phylogenetic Diversity

As previously mentioned, Feye et al. [13] investigated the microbiome response of the jejunum and ileum to the dietary inclusion of the microencapsulated blend of organic acids and botanicals. In that research, Feye et al. [13] demonstrated beta diversity differences in the jejunum between those fed diets supplemented with 0 and 500 g/MT. Similarly, the current study demonstrated these differences within the control treatments (non-infected). However, there were no differences between the supplementation 0 and 500 g/MT of the microencapsulated blend of organic acids and botanicals when under clinical NE infection. The lack of significance between the NE-treated groups was most likely due to the severely diseased state of the birds.

In the industry, NE will often present itself as a sub-clinical infection where antibiotics and alternatives have demonstrated a level of protection [41]. Under sub-clinical conditions, Emami et al. [42] demonstrated improved health (reduced lesion scores) and maintained growth performance by modifying the gut microbiome, tight junctions, immune response, and cell metabolism. In a companion study, Swaggerty [15] made it evident that the supplementation of a microencapsulated blend of organic acids and botanicals could potentially reduce clinical manifestations of NE by altering specific immune-related pathways under NE clinical conditions, as revealed by kinome analysis. Although immune-related pathways were changed [15], the clinical presentation of NE may have suppressed the abundance and phylogenetic response by reducing the overall abundance of the microbiome.

### 4.3. Necrotic Enteritis Narrows Core Microbiome

In the past, numerous efforts have been placed on determining the microbiome of poultry during infection with sub-clinical and clinical NE; however, much of the research has focused narrowly on the cecal microbiome [43,44,45,46]. As the primary site of infection is the small intestines, typically the ileum [47], it may be more imperative to delineate the microbial changes occurring there rather than the hindgut, the ceca. More recent work has focused on the ileum, where clinical damage occurs [48,49]. The current study identified the core microbiome under non-challenged and NE clinical conditions. Clinical NE narrows the core communities within the small intestines. The non-challenged birds fed 0 and 500 g/MT had 10 and 6 different ASVs prevalent in more than 50% of the samples, whereas those challenged narrowed to 5 and 7 core ASVs. The narrowing of the microbiome is further evident by the taxonomic profiles (Figure 5) and the significantly different phyla and genera. As *Clostridiaceae* increases among the NE-challenged broiler breeders, the taxa belonging to *Actinobacteriota*, *Bacteroidota*, and *Verrucomicrobiota* decrease (Figure S1). These communities are typically present among healthy broilers and are a sign of a healthy microbiome [50]. It is important to note that the treatment of 500 g/MT of the microencapsulated blend of organic acids and botanicals demonstrated a slight rebound in the abundance of these communities compared to those challenged with NE and fed 0 g/MT diets. The slight rebound could be attributed to the nature of the supplementation of the microencapsulated blend of organic acids and botanicals to select for *Lactobacillus* and *Clostridiaceae,* as seen by Feye et al. [13] and the current study.

### 4.4. Lactobacillus and Clostridiaceae Core ASVs of Microencapsulated Blend

In the current study, *Lactobacillus* and *Clostridiaceae* were identified as the core members of the microbiome of the unchallenged birds fed diets containing 500 g/MT but not in those fed diets containing 0 g/MT of the microencapsulated blend of organic acids and botanicals. The same core *Clostridiaceae* ASVs were also present in the 500 g/MT of the challenged group as in the 500 g/MT unchallenged group (dc85940d84ddbd7315db16b14390cb8d). The challenged group fed diets containing 500 g/MT also had an additional unique ASV belonging to *Clostridiaceae* (9dece060ffcff93473a7023dcee0defd). Similarly, past research investigating the effects of the same microencapsulated blend of organic acids and botanicals led to an increase in *Lactobacillus* and *Clostridiaceae* in the jejunum of birds fed 300 and 500 g/MT compared to those fed diets containing no supplements [13]. Similar products have also shown increased specific taxa belonging to *Lactobacillus* [40]. Pham et al. demonstrated increased *Lactobacillus* abundance in the ceca when non-challenged NE birds were fed diets containing 500 mg/kg of a microencapsulated blend of organic acids and botanicals compared to those provided the basal control diets.

Specific *Clostridial* species have been identified as probiotic candidates, as not all *Clostridium* are created equal [51]. Additionally, taxa belonging to the family of *Clostridiaceae* are recognized as members of a normal poultry microbiome [52]. As such, the taxa belonging to the family *Clostridiaceae* identified as the core microbiome members of those fed diets supplemented with 500 g/MT of the microencapsulated blend of organic acids and botanicals in the current study could be members of a healthy or normal microbiome even when infected with other *Clostridium* spp. to induce NE. Stanley et al. [53] correlated the feed conversion ratio (FCR) of broilers with the microbiome composition. They determined the presence of taxa belonging to the family *Clostridiaceae* was positively related to improved FCR [53]. Future studies would need to be conducted to narrow in on what specific species belonging to the family of *Clostridiaceae* is being selected by supplementing a microencapsulated blend of organic acids and botanicals.

## 5. Conclusions

The current results demonstrated the modulation of the small intestine microbiome by supplementing a microencapsulated blend of organic acids and botanicals. Specifically, this research demonstrated that the supplementation of the microencapsulated blend did not alter the richness and evenness of the microbiome when under a clinical challenge of NE but did impact the core microbiome and the differentially abundant taxa. The supplementation of the microencapsulated blend selected for *Lactobacillus* and *Clostridiaceae* among the core microbiome regardless of challenge conditions. As the specific ASVs belonging to the family *Clostridiaceae* were present regardless of NE infection, the supplementation of the microencapsulated blend could select for beneficial members of *Clostridiaceae* that were indicative of a more stable microbiome. Future research would be needed to better identify the specific *Clostridiaceae* to verify its functionality within the poultry GIT.

Under clinical NE conditions, the effects of the supplementation of the microencapsulated blend of organic acids and botanicals were masked to a certain degree. The NE infection dominated the small intestines, as indicated by the abundance of *Clostridiaceae* in the NE-challenged broiler breeders. As subclinical infection of NE is very common in the poultry industry, it would be beneficial to delineate the impacts of this microencapsulated blend on maintaining performance and intestinal microbiome balance.

## Figures and Tables

**Table 1:** Core microbiome (present in >50% of samples) of digesta of control and NE-induced birds.

Infection	Diet	Organisms	ASV
Control	0 g/MT	*Lachnospiraceae* (2 unique ASVs)	10650515e35dcecde52015f8c34a4a06
		*Lachnospiraceae_Blautia*	0b6c85919018ef580a0e1111b794c86b
		*Lachnospiraceae_Blautia_Lachnospiraceae_bacterium*	74e7c89ab2da511c0003f650773f9c2f
		*Lachnospiraceae*	492f2e502cce64557efbfad55333f9ca
		*Erysipelotrichaceae_Ileibacterium_Ileibacterium_valens*	a922583a2cd922f6e2361c1df16702bb
		*Enterobacteriaceae*	20cfe7f61d18f6525cc71caae0ab28dc
		*Sutterellaceae_Parasutterella*	cf39b94723be8d7c4e5b41f4b20dab44
		*Akkermansiaceae_Akkermansia*	b6b05223adf86d071fd279f79dc2533c
		*Peptostreptococcaceae*	ee59a70b1b832493583e7bc2d6488ec7
		*Peptostreptococcaceae*	931dbe33cb8438cfd863ac9acfb5d17f
		
	500 g/MT	*Lachnospiraceae_Blautia*	0b6c85919018ef580a0e1111b794c86b
		*Lachnospiraceae_*	492f2e502cce64557efbfad55333f9ca
		*Clostridiaceae_*	dc85940d84ddbd7315db16b14390cb8d
		*Lactobacillaceae_Lactobacillus*	44d9fc4de8898b6c82c69654435a9f0b
		*Enterobacteriaceae_*	20cfe7f61d18f6525cc71caae0ab28dc
		*Peptostreptococcaceae_*	931dbe33cb8438cfd863ac9acfb5d17f
			
NE-Induced	0 g/MT	*Clostridiaceae*	3be4ebb35ff7f6217fae945cb0ad5413
		*Clostridiaceae*	2acc5f779e35ac658d8389859048ff61
		*Clostridiaceae*	dc85940d84ddbd7315db16b14390cb8d
		*Clostridiaceae*	3b55963ebca3b90a7dcf9a27ef40a76a
		*Enterobacteriaceae*	20cfe7f61d18f6525cc71caae0ab28dc
		
	500 g/MT	*Clostridiaceae*	3be4ebb35ff7f6217fae945cb0ad5413
		*Clostridiaceae*	2acc5f779e35ac658d8389859048ff61
		*Clostridiaceae*	9dece060ffcff93473a7023dcee0defd
		*Clostridiaceae*	dc85940d84ddbd7315db16b14390cb8d
		*Clostridiaceae*	e52b990e8b8ae704003a5ff38b791dbf
		*Clostridiaceae*	3b55963ebca3b90a7dcf9a27ef40a76a
		*Enterobacteriaceae*	20cfe7f61d18f6525cc71caae0ab28dc

## Data Availability

Demultiplexed sequencing reads are available on NCBI Sequence Read Archive (PRJNA899529) and GitHub (https://github.com/RickeLab-UW/Microencapsulated-Blend-on-Broiler-Intestinal-Microbiota, accessed on 3 May 2023). As well, the QIIME2 metadata file is available on Git Hub. Please contact S.C.R. if you require additional information.

## Supplementary Materials

The following supporting information can be downloaded at: https://www.mdpi.com/article/10.3390/ani13101627/s1, Table S1: Main effect and interaction of treatment and trial on alpha diversity using ANOVA; Table S2: Effect of treatment and trial on the alpha diversity of broiler digesta; Table S3: Relating lesions scores to evenness and richness of the microbiome of commercial broilers; Table S4: Main effect and interaction of treatment and trial on the beta diversity of broiler digesta when using ADONIS; Table S5: Effect of treatment and trial on the beta diversity of broiler digesta; Figure S1: Different phyla impacted by the supplementation of a blend of microencapsulated organic acids and botanicals; Figure S2: Different taxa at the genus level, impacted by the supplementation of a blend of microencapsulated organic acids and botanicals.
