# Supplementary material for: Impact of a Blend of Microencapsulated Organic Acids and Botanicals on the Microbiome of Commercial Broiler Breeders under Clinical Necrotic Enteritis
Source: Animals (Basel). 2023 May 12;13(10):1627. doi: 10.3390/ani13101627 (PMC10215661; doi:10.3390/ani13101627)
Supplement: Supplementary file 1 [file animals-13-01627-s001.zip › animals-2343987-supplementary.pdf]

animals MDPI

Supplemental Information

Impact of a Blend of Microencapsulated Organic Acids and Botanicals on the Microbiome of Commercial Broiler Breeders under Clinical Necrotic Enteritis

Table S1. Main effect and interaction of treatment and trial on alpha diversity using ANOVA.

		Shannon's Entropy			Pielou's Evenness		
	df[1]	SS[2]	F[3]	*p* value[4]	SS	F	*p* value
Treatment	3	113.141	21.895	**< 0.001**	0.904	15.433	**< 0.001**
Trial	2	7.205	2.091	0.130	0.020	0.523	0.594
Treatment × Trial	6	16.610	1.607	0.155	0.265	2.258	**0.045**
Residual	87	148.131			1.679		

[1]degrees of freedom (df), [2]Sum of Squares., [3]F value: ratio of mean squares., [4]Siginificance is denoted by bolded values ($p < 0.05$).

Table S2. Effect of treatment and trial on the alpha diversity of broiler digesta.

		Shannon's Entropy[1]			Pielou's Evenness[1]		
Treatment 1	Treatment 2	H[2]	*p* value[3]	Q-value[3]	H	*p* value	Q-value
0 g/MT CON (n = 28)	500 g/MT CON (n = 23)	0.046	0.830	0.830	2.092	0.148	0.148
	0 g/MT NE (n = 21)	28.880	**< 0.001**	**< 0.001**	21.218	**< 0.001**	**< 0.001**
	500 g/MT NE (n = 27)	19.755	**< 0.001**	**< 0.001**	15.256	**< 0.001**	**< 0.001**
500 g/MT CON (n = 23)	0 g/MT NE (n = 21)	11.570	**0.001**	**0.001**	9.372	**0.002**	**0.004**
	500 g/MT NE (n = 27)	8.731	**0.003**	**0.005**	5.818	**0.016**	**0.024**
0 g/MT NE (n = 21)	500 g/MT NE (n = 27)	1.478	0.224	0.269	3.383	0.066	0.079

[1]Alpha diversity main effect and pairwise differences were delineated using Kruskal-Wallis, [2]H-value: test statistic for Kruskal-Wallis, [3]Bolded values denote significance ($p < 0.05$; $Q < 0.05$)

Table S3. Relating lesions scores to evenness and richness of the microbiome of commercial broilers.

	Shannon's Entropy				Pielou's Evenness			
	Coef.	SEM	z	*p* value	Coef.	SEM	z	*p* value
Intercept	4.788	0.505	9.476	**< 0.001**	0.789	0.044	17.756	**< 0.001**
Lesions	−0.105	0.556	−0.189	0.850	−0.032	0.055	−0.576	0.565
Treatment	−0.243	0.086	−2.811	**0.005**	−0.032	0.008	−3.959	**0.000**
Lesions x Treatment	−0.016	0.079	−0.202	0.840	0.004	0.008	0.528	0.597
Group Var	0.943	2.902			0.014	0.128		
Group x Trial Cov	−0.195	1.185			−0.004	0.051		
Trial Var	0.041	0.477			0.001	0.021		

Table S4. Main effect and interaction of treatment and trial on the beta diversity of broiler digesta when using ADONIS.

		Jaccard Distance					Weighted Unifrac				
Effect	df[1]	SS[2]	MS[3]	F[4]	R[2]	*p* value[5]	SS	MS	F	R[2]	*p* value
Treatment	1	2.424	2.424	6.626	0.061	**0.001**	1.098	1.098	29.994	0.227	**0.001**
Trial	1	1.530	1.530	4.183	0.039	**0.001**	0.185	0.185	5.043	0.038	**0.002**
Treatment × Trial	1	1.132	1.132	3.094	0.029	**0.001**	0.125	0.125	3.417	0.026	**0.008**
Residuals	94	34.387	0.366		0.871		3.442	0.037		0.710	
Total	97	39.473			1.000		4.851			1.000	

[1]degrees of freedom (df), [2]Sum of Squares, [3]F value: ratio of mean squares, [4]Mean Square Error, [5]Siginificance is denoted by bolded values ($p < 0.05$)

Table S5. Effect of treatment and trial on the beta diversity of broiler digesta[1,2].

		Jaccard Distance			Weighted Unifrac		
**Treatment 1**	**Treatment 2**	**R[3]**	***p* value**	**Q-value**	**R**	***p* value**	**Q-value**
0 g/MT CON (n = 28)	500 g/MT CON (n = 23)	0.138	**0.003**	**0.004**	0.0946	**0.012**	**0.0144**
	0 g/MT NE (n = 21)	0.418	**0.001**	**0.002**	0.5685	**0.001**	**0.0015**
	500 g/MT NE (n = 27)	0.307	**0.001**	**0.002**	0.4118	**0.001**	**0.0015**
500 g/MT CON (n = 23)	0 g/MT NE (n = 21)	0.428	**0.001**	**0.002**	0.49	**0.001**	**0.0015**
	500 g/MT NE (n = 27)	0.340	**0.001**	**0.002**	0.3616	**0.001**	**0.0015**
0 g/MT NE (n = 21)	500 g/MT NE (n = 27)	0.008	0.306	0.306	−0.0284	0.832	0.832

[1]Pairwise differences were delineated using ANOSIM, [2]Significance is denoted as bolded ($p < 0.05$; $Q < 0.05$); while trends are italicized ($p < 0.10$), [3]R, the ANOSIM statistic, compares mean ranked dissimilarities between groups to mean ranked dissimilarities within groups

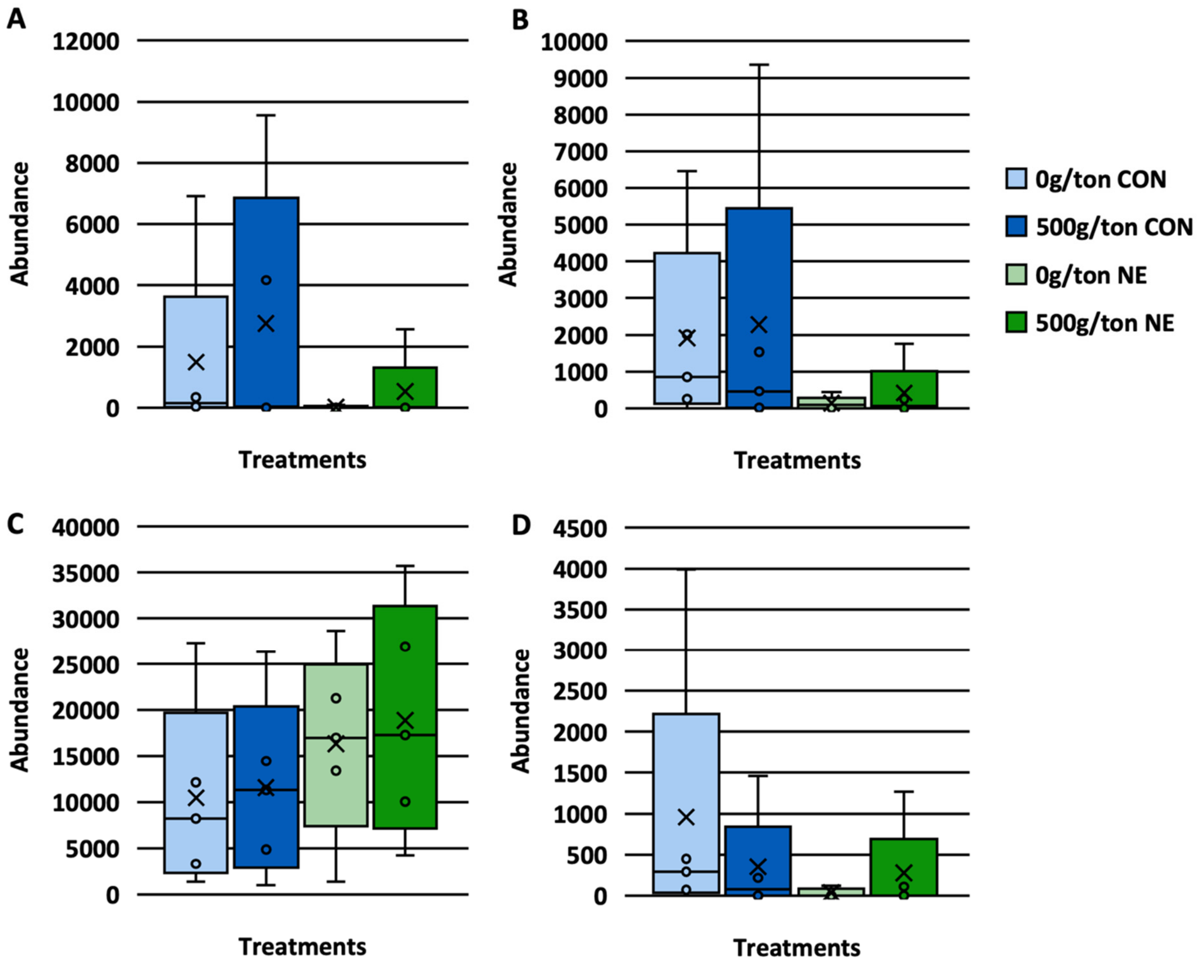

Figure S1. Significant different phyla, *Actinobacteriota* (**A**), *Bacteroidota* (**B**), *Firmicutes* (**C**), and *Verrucomicrobiota* (**D**), impacted by the supplementation of a blend of microencapsulated organic acids and botanicals (W = 24, 24, 26, 22; $p < 0.05$).

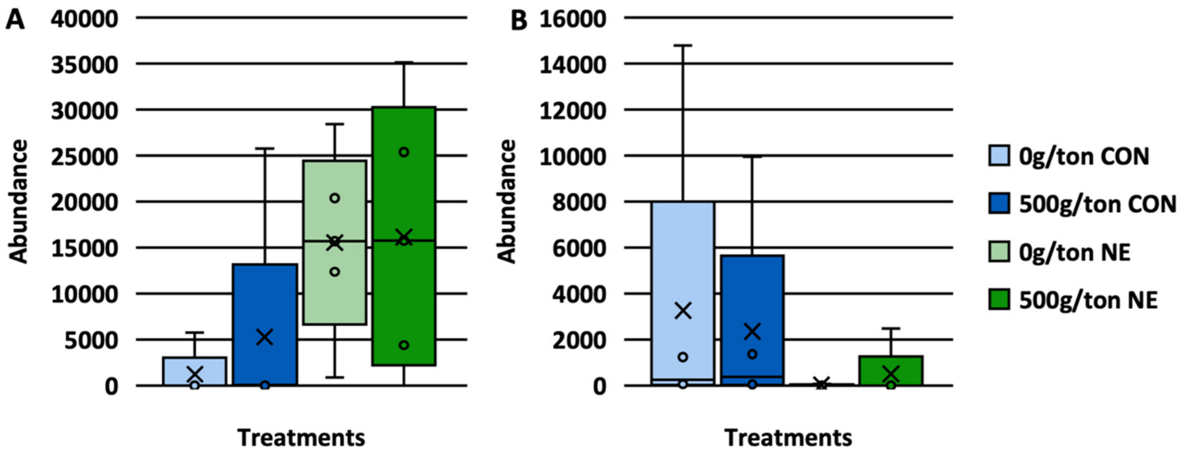

Figure S2. Significant different taxa at the genus level, *Clostridiaceae* (**A**) and *Peptostreptococcaceae* (**B**), impacted by the supplementation of a blend of microencapsulated organic acids and botanicals (W = 385, 369; $p < 0.05$).
